# Effectiveness of Electroacupuncture and Electroconvulsive Therapy as Additional Treatment in Hospitalized Patients With Schizophrenia: A Retrospective Controlled Study

**DOI:** 10.3389/fpsyg.2019.02306

**Published:** 2019-10-15

**Authors:** Jie Jia, Jun Shen, Fei-Hu Liu, Hei Kiu Wong, Xin-Jing Yang, Qiang-Ju Wu, Hui Zhang, Hua-Ning Wang, Qing-Rong Tan, Zhang-Jin Zhang

**Affiliations:** ^1^Department of Psychiatry, Xi’an Mental Health Center, Xi’an, China; ^2^School of Chinese Medicine, LKS Faculty of Medicine, The University of Hong Kong, Hong Kong, China; ^3^Department of Psychiatry, Xijing Hospital, The Fourth Military Medical University, Xi’an, China

**Keywords:** electroconvulsive therapy (ECT), acupuncture, schizophrenia, positive symptoms, negative symptoms, weight gain

## Abstract

Electroacupuncture (EA) and electroconvulsive therapy (ECT) are often used in the management of schizophrenia. This study sought to determine whether additional EA and ECT could augment antipsychotic response and reduce related side effects. In this retrospective controlled study, 287 hospitalized schizophrenic patients who received antipsychotics (controls, *n* = 50) alone or combined with EA (*n* = 101), ECT (*n* = 55) or both (EA + ECT, *n* = 81) were identified. EA and ECT were conducted for 5 and 3 sessions per week, respectively, with a maximum of 12 sessions for ECT during hospitalization. The Scale for the Assessment of Positive Symptoms (SAPS) and Scale for the Assessment of Negative Symptoms (SANS) were used to assess the severity of psychotic symptoms. Clinical response on SAPS and SANS, weight gain, and adverse events were compared. Survival analysis revealed that the ECT and EA + ECT groups had markedly greater clinical response rate than controls on SAPS [72.7 and 90.1% vs. 64.0%; relative risk (RR), 1.974 and 2.628, respectively, *P* ≤ 0.004] and on SANS (67.3 and 70.4% vs. 42.0%; RR, 1.951 and 2.009, respectively, *P* ≤ 0.015). A significantly greater response rate on SANS than controls was also observed in the EA group (64.4% vs. 42.0%; RR = 1.938, *P* = 0.008). EA-containing regimens remarkably reduced weight gain and incidences of headache, insomnia, dry mouth, and electrocardiographic abnormalities. These results suggest that EA and ECT can serve as additional treatment for enhancing antipsychotic response and reduce the side effects in hospitalized patients with schizophrenia.

**Clinical Trial Registration:** http://www.chictr.org.cn/showprojen.aspx?proj=38901, identifier ChiCTR1900023563.

## Introduction

Schizophrenia is a severe and highly disabling mental disorder that affects about 0.3% of the world population (Charlson et al., 2018). With antipsychotic drugs as the first-line treatment of schizophrenia, a large portion of the patients could not achieve satisfactory outcomes and develop relapse or become a chronic condition (Lally and MacCabe, 2015). Long-term antipsychotic treatment also often lead to weight gain and other side effects, which possibly explains low medication adherence and poor outcomes (Zhao et al., 2016). Different antipsychotic drugs have been demonstrated to be differentially associated with weight gain (Leucht et al., 2013). These have led to an increasing desire of seeking non-pharmacological alternatives that could enhance efficacy and reduce related side effects.

Since electroconvulsive therapy (ECT) was introduced in 1950s, it has been widely used in the treatment of psychotic disorders (Ali et al., 2019; Grover et al., 2019). It is well documented that ECT is remarkably effective in rapidly relieving positive symptoms (Ali et al., 2019; Grover et al., 2019). Over the past two decades, ECT also has been increasingly utilized in the clinical practice of psychiatry in China, where ECT often serves as adjunctive therapy with antipsychotic drugs (Tang et al., 2012). One recent meta-analysis suggests that additional ECT has a positive effect on mid-term clinical response for patients with treatment-resistant schizophrenia (Sinclair et al., 2019). However, ECT is frequently associated with adverse effects, such as headache and transient global amnesia (Andrade et al., 2016).

As a highly recognized alternative therapy, acupuncture has been widely used in the clinical practice of psychiatry in China and other East Asian countries (Pilkington, 2013). Numerous studies have suggested that acupuncture has multiple benefits in treating psychiatric symptoms, particularly insomnia, anxiety, and depression (Pilkington, 2013). Add-on acupuncture has beneficial effects in improving the positive, negative, cognitive symptoms, and the accompanying sleep disorders in patients with schizophrenia (Shen et al., 2014; Bosch et al., 2015; van den Noort et al., 2018). Acupuncture is also effective in controlling weight gain (Zhang R. Q. et al., 2017; Kim et al., 2018) and reducing antipsychotic-associated side effects (Shen et al., 2014).

We hypothesized that ECT and acupuncture could augment antipsychotic efficacy and reduce the side effects in patients with schizophrenia. To test this hypothesis, a retrospective controlled study was conducted to determine whether additional electroacupuncture (EA) and ECT, either used in separate or in combination, could produce better outcomes than antipsychotics alone in improving psychotic symptoms and reducing side effects in patients with schizophrenia.

## Materials and Methods

### Setting and Subjects

This retrospective study was conducted in the Xi’an Mental Health Center which is a public psychiatric hospital in Xi’an, Shaanxi, China, and has been registered in Chinese Clinical Trial Register (ChiCTR1900023563)^1^. The Xi’an Mental Health Center provides comprehensive services for patients with various psychiatric disorders. In November 2014, the Center has established a fully accessible and searchable electronic medical record system. This study then targeted and screened patients who were hospitalized between November 30, 2014 and December 1, 2018 in the Department of Early Intervention-I of the Center.

Patients were included in this study if they: (1) were either gender aged 18–75 years; (2) were hospitalized and diagnosed with chronic schizophrenia according to the International Classification of Diseases (10th version) (ICD-10) (World Health Organization [WHO], 2014); (3) had antipsychotic treatment comprising olanzapine or risperidone combined with or without EA or/and ECT during their stay in the hospital; and (4) had complete medical record and full clinical assessment.

Patients were excluded from the study if they had: (1) other nerve and brain stimulation therapies besides EA and ECT during hospitalization; (2) acupuncture treatment for 1 week or longer before admission to hospital; (3) a history of brain tumors or intracranial space-occupying lesions; (4) a history of substance abuse over the last one year; or (5) investigational treatments over the last year.

### Antipsychotic Treatment

A large proportion of patients had received treatment with olanzapine- and risperidone-containing regimens during their stay in the hospital. These two drugs are commonly prescribed antipsychotics which have been recommended as first-line agents for the management of schizophrenia in China (Li et al., 2015). Olanzapine and risperidone doses generally commenced at 5 and 2 mg/day, and gradually increased to an optimal dose within 1 week, but limited to 20 and 8 mg/day, respectively. Other antipsychotic drugs, such as ziprasidone, aripiprazole, haloperidol, and mood stabilizers, anxiolytics, and hypnotics were also often prescribed as additional treatment at the psychiatrist’s discretion, depending on patient’s condition and response. For those who had significant agitation, hostility, and aggressive behavior, haloperidol injection was immediately given in combination with ECT. Previous studies have confirmed that haloperidol injection had particular effects in managing hostile and aggressive acutely schizophrenic patients and agitation (Tuason, 1986; Bieniek et al., 1998).

### ECT Intervention

While whether patients had ECT treatment was at the discretion of a psychiatrist, depending on the severity of positive symptoms and patients’ condition, ECT was immediately administered in combination with haloperidol if patients had significant agitation, hostility, or aggressive behavior at their admission to hospital. ECT has been used for acute management of schizophrenia with high aggression risk at time of admission in China (Zhang Q. E. et al., 2016). ECT was conducted for 3 sessions per week, but the total number should not exceed 12 sessions during hospitalization.

Patients were asked to have at least 8 h of fasting prior to ECT. For the ECT procedure, firstly patients received one dose of 0.5 mg atropine to reduce salivation and respiratory tract secretions, with one dose of 1–2 mg/kg propofol for anesthesia, followed by one dose of muscle relaxant succinylcholine (1–2 mg/kg). Subsequently ECT was performed on a spECTrum 5000Q machine (MECTA Corporation, United States) with the placement of stimulus electrodes on the right temple and the vertex of the scalp as previously reported (Nobler and Sackeim, 2008). The initial stimulus intensity (percentage of energy) was set at 5% equivalent to 25 mV with a fixed constant current of 0.9 A, and then gradually increased until a seizure discharge that lasted for a minimum of 20 s, as indicated in electroencephalogram (EEG) was achieved.

### Electroacupuncture Treatment

Electroacupuncture has become a standard therapy provided to hospitalized patients at the Center. The treatment was carried out by acupuncturists at bedside for 5 sessions per week (once per day in weekdays). EA protocol for schizophrenia has been well established at the Center. Briefly, the following acupoints were used: Shen-Ting (GV24), Bai-Hui (GV20), Si-Shen-Cong (EX-HN1), Feng-Chi (GB20), Shen-Men (HT7), and Nei-Guan (P6). Disposable acupuncture needles (0.30 mm in diameter, 25–40 mm in length) were used for EA. The needles were penetrated 0–30 mm in depth at acupoints in a perpendicular or oblique direction. Manual manipulation was then carried out until the patients felt needling sensation (De-Qi). Electrical stimulation was then applied with connections between Shen-Ting (GV24) and Bai-Hui (GV20) and between left and right Feng-Chi (GB20). The output peak current and voltage of the machine (G6805-A electrical stimulator) were 6 V and 48 mA, respectively. Stimulus parameters were constant wave at frequency of 100 Hz and phase duration of 100 μs for 20 min. The intensity of stimulation was gradually increased to a level at which patients obtained considerable electric stimulus sensation.

### Clinical Assessments

The Scale for the Assessment of Positive Symptoms (SAPS, Chinese version) (Andreasen, 1984) and the Scale for the Assessment of Negative Symptoms (SANS, Chinese version) (Andreasen, 1989) are routine clinical instruments used for the assessment of the severity of schizophrenia at the Center. The assessment was done once weekly during patients’ hospitalization by designated psychiatrists who had received a series of training workshops to ensure assessment consistency and reliability. The Center held training workshops biannually. The primary outcome was the clinical response, which is defined as an at least 30% reduction from baseline in score on SAPS and SANS on a weekly basis. The clinical response rate was compared over time in the four groups using survival analysis (see below). Body weight gain was recorded on a weekly basis. Other side effects were recorded using the Treatment Emergent Symptom Scale (TESS) (Guy, 1976).

In China, the determination of discharge of patients from hospital was not only upon clinical remission of positive symptoms as defined previously (Lambert et al., 2010), but also upon administrative policies and patients’ individual socioeconomic situation. Hospital stay days could not accurately reflect clinical remission and were therefore not included as a clinical outcome, but served as a covariate for outcome analysis (see below).

### Data Analysis

Our previous study has shown that additional ECT could produce an approximately 25% greater clinical response rate than antipsychotics alone in acute treatment of psychotic episode (Zhang et al., 2012a). In this study, a sample size of 287 with an average 72 per group could yield at least an 80% power to detect a 25% difference in the clinical response rate at a significant level of 0.05 between any two groups.

Categorical baseline parameters, antipsychotic regimens, and incidence of adverse events were analyzed using Chi-square (χ^2^) test. Continuous baseline data was analyzed using one-way analysis of variance (ANOVA), followed by Student–Newman–Keuls method to further detect between-group differences. Net weight gain was analyzed using analysis of covariance (ANCOVA) with age, duration of the illness, number of previous psychotic episodes, number of previous hospitalizations, and current hospital stay days as covariates. Whether patients were treated with a particular antipsychotic drug with which proportion of patients had significant differences among the four groups also served a covariate in the analysis of weight gain.

Despite the fact that hospital stay days largely varied, the majority of patients had achieved clinically meaningful improvement, stable partial, or even full remission within 14 weeks. Therefore, Week 14 was chosen as the endpoint for survival analysis. Cox regression proportional hazards model was used for survival analysis to examine differences in time to clinical response at 14 weeks among the four groups with adjustment for age, duration of the illness, number of previous psychotic episodes, number of previous hospitalizations, and current hospital stay days. Statistical significance was defined as a two-sided *P*-value of <0.05. The analyses were performed with SPSS version 19 software (Chicago, IL, United States).

## Results

### Baseline Characteristics of Patients

**FIGURE 1:**
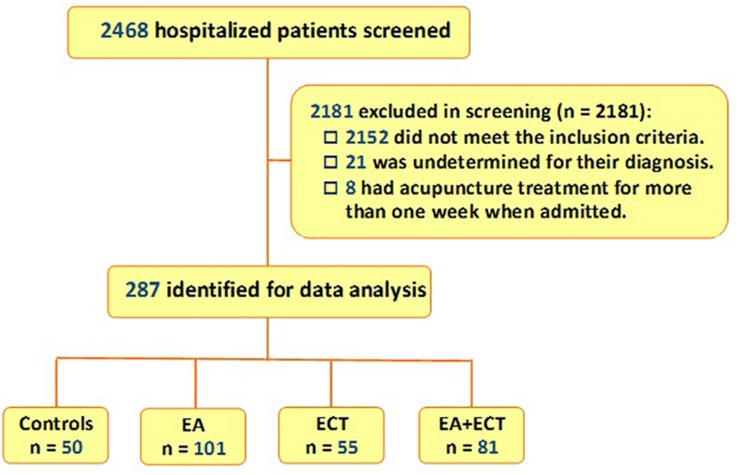
Flowchart of selection of study subjects from schizophrenic patients who were hospitalized in Department of Early Intervention-I of the Xi’an Mental Health Center at Xi’an, China, between November 30, 2014 and December 1, 2018. EA, electroacupuncture; ECT, electroconvulsive therapy.

Of 2,468 patients who were hospitalized in the Department of Early Intervention-I of the Center between November 30, 2014 and December 1, 2018, 287 met the inclusion criteria and were included in the study. While all had antipsychotic treatment, 50 were treated with pharmacotherapy alone; 101 and 50 received additional EA and ECT, respectively; other 81 had a combination of EA and ECT (Figure 1).

Baseline characteristics are summarized in Table 1. Significant differences among the four groups were observed on age, duration of the illness, number of previous psychotic episodes, number of previous hospitalization, and current hospital stay days. These variables served as covariates in survival analysis and weight gain analysis. Other baseline variables were not different among the four groups. The severity of psychotic symptoms measured with baseline SAPS and SANS scores and other baseline variables were not significantly different.

**TABLE 1:** Baseline characteristics^a^.

**Variables**	**Control (*n* = 50)**	**EA (*n* = 101)**	**ECT (*n* = 55)**	**EA + ECT (*n* = 81)**	***P*-value**
Age, year^b^	39.7 ± 13.7	40.2 ± 12.7	33.3 ± 9.6	36.3 ± 11.3	0.003
Gender, *n* (%)^c^					0.123
Male	22 (44.0)	51 (50.5)	17 (30.9)	38 (46.9)	
Female	28 (56.0)	50 (49.5)	38 (69.1)	43 (53.1)	
Residential areas, *n* (%)^c^					0.317
Urban and town	26 (52.0)	57 (56.4)	23 (41.8)	38 (46.9)	
Rural	24 (48.0)	44 (43.6)	32 (58.2)	43 (53.1)	
Educational degree, *n* (%)^c^					0.183
Primary and illiteracy	10 (20.0)	13 (12.9)	10 (18.2)	12 (14.8)	
Secondary	30 (60.0)	68 (67.3)	25 (45.5)	47 (58.0)	
College and above	10 (20.0)	20 (19.8)	20 (36.4)	22 (27.2)	
Employment status, *n* (%)^c^					0.251
On work	19 (38.0)	34 (33.7)	29 (52.7)	35 (43.2)	
Unemployed/retired	27 (54.0)	61 (60.4)	21 (38.2)	42 (51.9)	
Students	4 (8.0)	6 (5.9)	5 (9.1)	4 (4.9)	
Marital status, *n* (%)^c^					0.070
Single/divorce/widow	29 (58.0)	43 (42.6)	26 (47.3)	49 (60.5)	
Married	21 (42.0)	58 (57.4)	29 (52.7)	32 (39.5)	
Family history with mental disease, *n* (%)^c^	13 (26.0)	31 (30.7)	19 (34.5)	16 (19.8)	0.223
Duration of the illness, year^b^	11.5 ± 10.4	13.9 ± 10.6	7.3 ± 7.2	8.0 ± 8.2	<0.001
no. of previous psychotic episodes^b^	3.4 ± 4.1	4.3 ± 4.5	2.9 ± 1.7	2.5 ± 1.9	0.002
no. of hospitalization^b^	3.7 ± 4.2	4.6 ± 4.4	2.9 ± 2.1	2.8 ± 2.3	0.005
Current hospital stay days^b^	41.9 ± 21.8	56.1 ± 27.1	38.1 ± 13.1	53.6 ± 19.7	<0.001

*^*a*^EA, electroacupuncture; ECT, electroconvulsive therapy. ^*b*^Continuous data are expressed mean ± SD and were examined using one-way analysis of variance (ANOVA). ^*c*^Categorical data were examined using Chi-square (χ^2^) test.*

### Psychotropic Medication Modes

The five most commonly prescribed antipsychotic drugs were olanzapine(75.6%, 217/287), risperidone (30.0%, 86/287), haloperidol (20.6%, 59/287), ziprasidone (18.1%, 52/287), and aripiprazole (5.6%, 16/287) (Table 2). The EA + ECT group had a markedly higher proportion of patients who were treated with haloperidol than the other three groups (*P* = 0.003) and most of them received haloperidol injection. Whether patients received haloperidol treatment then served an additional covariate in the analysis of weight gain. There were 56.1% (161/287) patients receiving antipsychotic combination regimens. No significant differences were detected on a proportion of patients who received mono- and combination therapy with antipsychotics among the four groups (Table 2).

**TABLE 2:** Antipsychotic regimens used in patients with schizophrenia^a,b^.

	**Control**	**EA**	**ECT**	**EA + ECT**	
**Antipsychotics**	**(*n* = 50)**	**(*n* = 101)**	**(*n* = 55)**	**(*n* = 81)**	***P*-value**
**5 most commonly used antipsychotics**					
Olanzapine	36 (72.0)	73 (72.3)	43 (78.2)	65 (80.2)	0.551
Risperidone	17 (34.0)	30 (29.7)	14 (25.5)	25 (30.9)	0.812
Ziprasidone	10 (20.0)	14 (13.9)	16 (29.1)	12 (14.8)	0.093
Aripiprazole	4 (8.0)	6 (5.9)	3 (5.5)	3 (3.7)	0.772
Haloperidol ^c^	6 (12.0)	16 (15.8)	9 (16.4)	28 (34.6)	0.003
**Regimens**					0.745
Monotherapy	24 (48.0)	44 (43.6)	26 (47.3)	32 (39.5)	
Combination therapy	26 (52.0)	57 (56.4)	29 (52.7)	49 (60.5)	

*^*a*^EA, electroacupuncture; ECT, electroconvulsive therapy. ^*b*^Data were examined using Chi-square (χ^2^) test. ^*c*^Most patients received haloperidol injection for their significant agitation, hostility, or aggressive behavior.*

### Clinical Response Rate

**FIGURE 2:**
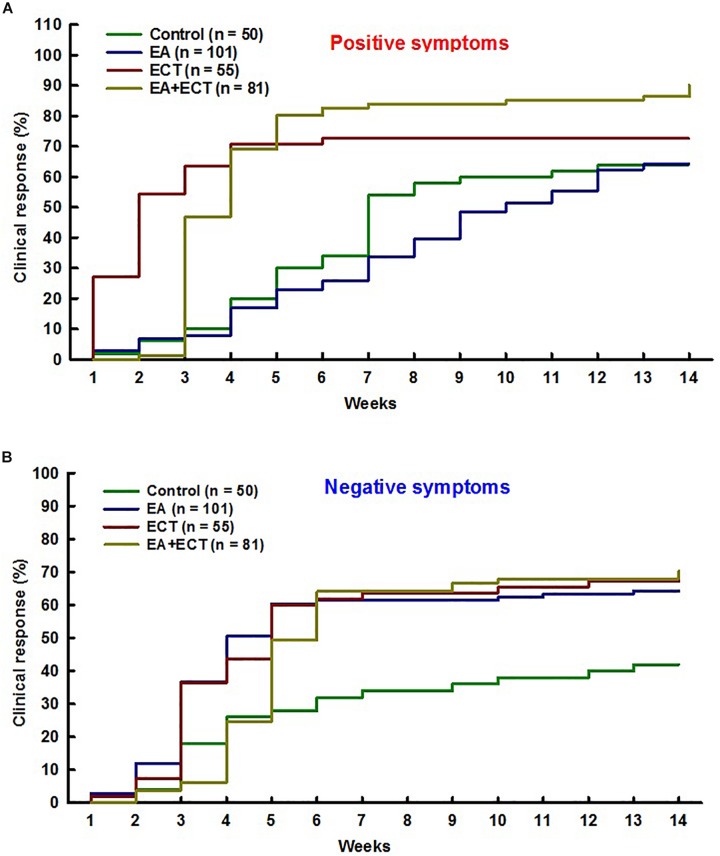
Cox regression proportional hazards model with relative risk (RR) was used for survival analysis on odds of achieving clinical response on positive symptoms **(A)** and negative symptoms **(B)** over 14 weeks of hospitalization of schizophrenic patients treated with additional electroacupuncture (EA), electroconvulsive therapy (ECT), or a combination of both (EA + ECT). The analysis was adjusted for age, duration of the illness, number of previous psychotic episodes, number of previous hospitalization, and current hospital stay days. For positive symptoms, EA vs. control: RR = 0.931, *P* = 0.742; ECT vs. control: RR = 1.974, *P* = 0.004; EA + ECT vs. control: RR = 2.628, *P* = 0.000; ECT vs. EA: RR = 2.120, *P* = 0.000; EA + ECT vs. EA: RR = 2.823, *P* = 0.000; EA + ECT vs. ECT: RR = 1.331, *P* = 0.149. For negative symptoms, EA vs. control: RR = 1.938, *P* = 0.008; ECT vs. control: RR = 1.951, *P* = 0.015; EA + ECT vs. control: RR = 2.009, *P* = 0.006; ECT vs. EA: RR = 1.007, *P* = 0.975; EA + ECT vs. EA: RR = 1.037, *P* = 0.844; EA + ECT vs. ECT: RR = 1.030, *P* = 0.890.

Survival analysis revealed significant differences among the four groups in clinical response rate on SAPS (*P* = 0.000) and SANS (*P* = 0.037) over 14 weeks (Figure 2).

For SAPS, additional ECT and EA + ECT produced a significantly greater response rate (72.7 and 90.1%) compared to the control group (64.0%) with relative risk (RR) of 1.974 (*P* = 0.004) and 2.628 (*P* = 0.000), respectively. The response rate of both ECT and EA + ECT regimens did not differ with a RR of 0.751 (*P* = 0.149), but was markedly higher than that of additional EA (64.4%) with RR of 2.120 and 2.823 (*P* = 0.000), respectively. The response rate of additional EA and controls was not different with a RR of 0.971 (*P* = 0.742).

For SANS, all the three additional treatment groups (EA, ECT, and EA + ECT) displayed a markedly greater response rate than the control group (64.4, 67.3, and 70.4% vs. 42.0%) with RR of 1.938 (*P* = 0.008), 1.951 (*P* = 0.015), and 2.009 (*P* = 0.006), respectively. The response rate of the three additional regimens was not different.

### Net Weight Gain

**FIGURE 3:**
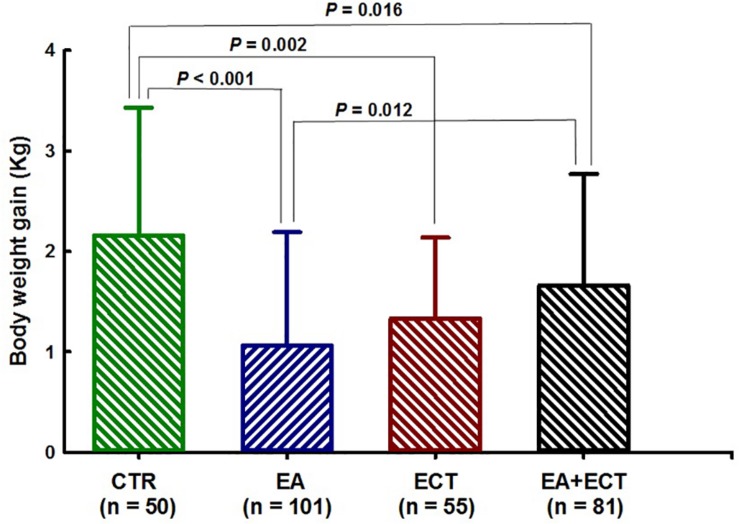
Effects of additional electroacupuncture (EA), electroconvulsive therapy (ECT), or a combination of both (EA + ECT) on net weight gain of hospitalized patients with schizophrenia. Data are expressed mean ± SD and were analyzed using analysis of covariance (ANCOVA) with age, duration of the illness, number of previous psychotic episodes, number of previous hospitalization, current hospital stay days, and whether patients received haloperidol treatment as covariates, followed by Student–Newman–Keuls method to further detect between-group differences.

Net weight gain was compared among the four groups with adjustment for age, duration of illness, number of previous psychotic episodes and hospitalization, current hospital stay days, and whether patients received haloperidol (Figure 3). ANCOVA revealed a significant difference (*F* = 9.383, *P* < 0.001). An average weight gain (1.06 kg) of the EA group was markedly lower than that of the control group (2.16 kg, *P* < 0.001) and the EA + ECT group (1.65 kg, *P* = 0.012). Net weight gain of the control group (2.16 kg) was significantly greater than that of the ECT group (1.33 kg, *P* = 0.002) and the EA + ECT group (1.65 kg, *P* = 0.016).

#### Adverse Events

Table 3 summarizes side effects observed, with no significant differences in the overall incidence of adverse events among the four groups; but the ECT group had a much higher rate of headache than the other three groups (*P* = 0.002). The control and ECT groups exhibited higher incidence of ECG abnormalities (*P* < 0.001), insomnia (*P* = 0.014), and dry mouth (*P* = 0.011) compared to the two EA-containing groups.

**TABLE 3:** The incidence of adverse events^a,b^.

	**Control**	**EA**	**ECT**	**EA + ECT**	
	**(*n* = 50)**	**(*n* = 101)**	**(*n* = 55)**	**(*n* = 81)**	***P*-value**
Any	8 (16.0)	18 (17.8)	8 (14.5)	12 (14.8)	0.936
Headache	4 (8.0)	2 (2.0)	8 (14.5)	1 (1.2)	0.002
Decreased activity	8 (16.0)	5 (5.0)	4 (7.3)	12 (14.8)	0.062
Tremor	1 (2.0)	6 (5.9)	1 (1.8)	1 (1.2)	0.250
Akathisia	1 (2.0)	4 (4.0)	1 (1.8)	1 (1.2)	0.658
Heart pound	2 (4.0)	2 (2.0)	0	2 (2.5)	0.546
Muscle rigidity	3 (6.0)	1 (1.0)	2 (3.6)	1 (1.2)	0.223
Hypersalivation	1 (2.0)	2 (2.0)	0	1 (1.2)	0.758
Constipation	1 (2.0)	2 (2.0)	1 (1.8)	2 (2.5)	0.994
ECG abnormalities	19 (38.0)	14 (13.9)	15 (27.3)	10 (12.3)	<0.001
Insomnia	8 (16.0)	4 (4.0)	7 (12.7)	3 (3.7)	0.014
Dry mouth	6 (12.0)	1 (1.0)	4 (7.3)	2 (2.5)	0.011

*^*a*^EA, electroacupuncture; ECT, electroconvulsive therapy. ^*b*^Data were examined using Chi-square (χ^2^) test.*

## Discussion

The central theme of this retrospective controlled study was to systematically evaluate the effectiveness and safety profile of EA and ECT and a combination of both as additional treatment in hospitalized patients with schizophrenia. All subjects included in this study were experiencing a severe psychotic episode, with multiple previous episodes and an average illness duration of 7–14 years, suggesting that most patients’ condition was lingering and recurrent. The majority of patients were treated with olanzapine and risperidone in either monotherapy or combination regimens, as these two drugs are the most commonly prescribed antipsychotic agents in acute and long-term treatment of schizophrenia in China (Li et al., 2015). We noted that the EA + ECT group had a much higher proportion of patients who received additional haloperidol than the other three groups, with approximately 67% vs. 12–16%. This was because haloperidol injection was often combined with ECT for acute management of agitation, hostility, and aggressive behavior. ECT combined with haloperidol has been shown to induce a transient redistribution of haloperidol (Aoba et al., 1983). It appears that ECT may have effects on pharmacokinetic profile of haloperidol.

We found that the patients who received additional ECT-containing regimens had an approximately twofold to threefold greater odds of achieving clinical response on both positive and negative symptom domains compared to controls over the course of hospitalization; the greater improvement of most patients treated with ECT-containing regimens was observed as early as 1–4 weeks. These results clearly indicate that additional ECT can augment and accelerate antipsychotic response. This finding is highly consistent with a meta-analysis conclusion that a combination of ECT with antipsychotics could produce rapid global improvement and reduction of symptoms (Tharyan and Adams, 2005). Nevertheless, unlike previous studies in which ECT was generally used as a last option to treat refractory schizophrenia (Grover et al., 2018), in the present study, ECT was introduced immediately to antipsychotic regimens when patients were hospitalized. Our previous study also has confirmed that additional ECT augmented antipsychotic efficacy in adolescents with first-episode psychosis, without causing apparent side effects (Zhang et al., 2012a).

One meta-analysis has suggested that acupuncture may have some antipsychotic effects measured with global and mental state (Shen et al., 2014). This study further revealed that, like ECT, EA also had the augmenting effects in reducing negative symptoms, with an approximately twofold greater odds of achieving clinical response on SAPS compared to antipsychotic regimens alone, although it did not produce additional improvement on positive symptoms. Individuals with schizophrenia often experienced persistent negative symptoms, such as blunted mood, poverty of speech, apathy, anhedonia, and cognitive impairment (Veerman et al., 2017). Antipsychotic drugs are less effective and even ineffective in treating primary negative symptoms (Veerman et al., 2017). Nevertheless, it has been found that acupuncture could improve working memory of patients with chronic schizophrenia (Bosch et al., 2016; van den Noort et al., 2018). A large body of evidence confirms the effectiveness of EA in alleviating various depressive disorders (Zhang et al., 2010) and mild cognitive impairment (Deng and Wang, 2016). EA also has therapeutic effects against anhedonic behavior in animal models (Yu et al., 2007). These studies, together with this study, suggest that EA may have particular effects in reducing negative symptoms and comorbid psychiatric symptoms of schizophrenia. On the other hand, the EA + ECT group had approximately 17–26% higher response rate than the other two groups on positive symptoms, although some differences did not reach statistical significance level. It seems that EA combined with ECT could produce additive and even synergistic augmenting effects on positive symptoms. However, unlike positive symptoms, the clinical response rate of the EA + ECT group was similar to that of the EA and ECT groups on negative symptoms (between 64 and 70%). Additive effects of the two interventions seem least on negative symptoms, probably due to the fact that there exists a ceiling effect or the current length of treatment was not sufficient to produce additive effects on negative symptoms.

The possible mechanisms of acupuncture’s efficacy on schizophrenia may be related to its broad neuromodulation (Zhang et al., 2012b). Acupuncture could robustly modulate multiple catecholaminergic and neuropeptidergic neuronal systems, including dopamine, serotonin, noradrenaline, and endogenous opiate neuropeptides of the brain (Ulett et al., 1998). It also broadly affects brain regions associated with the pathogenesis of psychotic disorders (Dhond et al., 2007).

Weight gain is a prevalent and serious side effect of antipsychotic treatment (Zhang J. P. et al., 2016). In this study, the control group treated only with antipsychotic drugs gained approximately 2.2 kg of body weight during hospitalization. The addition of EA, however, reduced weight gain by approximately 0.3–1.1 kg as compared to the other three groups. The ECT and EA + ECT groups also gained less weight than the control group. These results demonstrated the effectiveness of both EA and ECT in controlling antipsychotic-related weight gain. Similar results also have been observed in acupuncture treatment of olanzapine-induced obesity (Zhang L. et al., 2017) and simple obesity (Zhang R. Q. et al., 2017; Kim et al., 2018). One case report also has shown that ECT reduced weight gain in a patient with comorbid severe obesity, binge-eating disorder, and bipolar disorders (Rapinesi et al., 2013). It is noted that, as mentioned above, the EA + ECT group had a much higher proportion of patients who received haloperidol treatment than the other three groups (34.6% vs. 12.0–16.4%). As a typical antipsychotic, haloperidol-induced weight gain seems less responsive to acupuncture treatment, probably due to its higher metabolic liability compared to second-generation antipsychotics (Solmi et al., 2017). This could explain the smaller amount of weight loss in the EA + ECT group than the EA group.

Post-ECT headache is a commonly occurring side effect (Haghighi et al., 2016). ECG abnormalities, insomnia, and dry mouth are often associated with antipsychotic treatment (Orsolini et al., 2016). In this study, we observed that the EA and EA + ECT groups had much lower incidences of headache, ECG abnormalities, insomnia, and dry mouth compared to the control and ECT groups. Previous studies also have shown acupuncture was effective in alleviating sleep disturbance, dry mouth, and tachycardia in patients with schizophrenia (Shen et al., 2014; Bosch et al., 2016; van den Noort et al., 2018). These results indicate that acupuncture has apparent benefits in treating ECT- and antipsychotic-related side effects. This also could partly explain the particular effects of EA on negative symptoms observed in this study.

In summary, additional ECT can augment antipsychotic effects against both positive and negative symptoms. Additional EA may have particular effects in reducing negative symptoms and antipsychotic- and ECT-related side effects, including antipsychotic-induced weight gain and ECT-related headache. We suggest that EA and ECT can serve as additional treatment for enhancing antipsychotic response and reduce side effects in hospitalized patients with schizophrenia.

## Limitations

Multiple limitations of this study should be noted. Firstly, as a retrospective controlled study, blinding was not established in psychiatrists and patients, their expectation on treatment outcomes might potentially interfered with data analysis. In addition, doses and treatment length of the targeted antipsychotics could not be well controlled, and there was a lack of the analysis of interrater reliability in this retrospective studies. Secondly, significant variations exist in multiple baseline variables, including age, duration of the illness, number of previous psychotic episodes, number of previous hospitalization, current hospital stay days, and proportion of patients who received haloperidol treatment. These variations suggest that older patients with longer hospital stays and more persistent and recurrent schizophrenia were more likely to receive acupuncture treatment. On the other hand, the subjects identified in this study represent a subpopulation of Chinese patients who may have distinctive perceptions of EA and ECT. Whether similar treatment outcomes of EA and ECT could be achieved in other subpopulations with schizophrenia needs further investigation. Thirdly, we did not develop sham EA and sham ECT procedure for controlled group. As EA and ECT to some extent may cause fear or other adverse experience, potential bias caused by the procedure-related negative psychological effects should be considered, although we expect that such negative effects would not have significant interference with treatment outcomes. Finally, the choice of acupuncture points used in this study was mainly based on traditional Chinese medicine (TCM) doctrine and empirical evidence. Acupuncture regimens used for the treatment of schizophrenia also varied from one to another (Shen et al., 2014). Various acupuncture regimens for schizophrenia should be optimized and standardized in the future.

## DATA AVAILABILITY STATEMENT

The datasets generated for this study are available on request to the corresponding author.

## Ethics Statement

The studies involving human participants were reviewed and approved by the Medical Ethical Committee of the Xi’an Mental Health Center. The patients/participants provided their written informed consent to participate in this study.

## Author Contributions

JJ and Z-JZ were involved in the conception and design of the study, data analysis, and preparation of the manuscript. JJ, JS, and F-HL collected the data. HW helped for the statistical analysis. X-JY helped for registration and data collection. Q-JW, HZ, H-NW, and Q-RT provided the critical comments.

## Conflict of Interest

The authors declare that the research was conducted in the absence of any commercial or financial relationships that could be construed as a potential conflict of interest.

## Footnotes

1 http://www.chictr.org.cn/showprojen.aspx?proj=38901
